# Technical Considerations in One Anastomosis Gastric Bypass—the Israeli Society of Metabolic and Bariatric Surgery Experience

**DOI:** 10.1007/s11695-024-07223-3

**Published:** 2024-04-23

**Authors:** Adam Abu-Abeid, Jonathan Benjamin Yuval, Andrei Keidar, Eran Nizri, Guy Lahat, Shai Meron Eldar, the ISMBS collaborative group, Shimon Sapojnikov, Zvi Howard Perry, Nissim Geron, Hasan Kais, Igor Waksman, Gideon Sroka, Jacob Rachmuth, Idan Carmeli, Kamal Khatib, David Goitein, David Hazzan, Yonatan Lessing, Boris Kirshtein, Shlomi Rayman, George Daud, Moaad Farraj, Asnat Raziel, Sa’d Sayida, Nasser Sakran, Mordechai Shimonov, Ronit Grinbaum, Dvir Froylich, Ahmad Assalia, Ahmad Mahajna, Mohamad Jazmawi, Anya Wexler Feigin, BADRAN BADRAN, Uri Kaplan, Joseph Kuriansky, Subhi Abu-Abeid, Igor Dashkovsky, Jamal Gazmawi, BISHARA BISHARA, Harbi Khalayleh, Moshe Rubin

**Affiliations:** Division of General Surgery, Tel Aviv Sourasky Medical Center, Sackler Faculty of Medicine, Tel Aviv University, 6 Weizman Street, 64230906 Tel Aviv, Israel

**Keywords:** One anastomosis gastric bypass, Technique, Biliopancreatic limb length, ISMBS

## Abstract

**Background:**

One anastomosis gastric bypass (OAGB) is gaining popularity worldwide due to its safety and effectiveness. OAGB is the most commonly performed metabolic bariatric surgery (MBS) in Israel. Israel is the only country where OAGB is the most prevalent MBS. Our aim is to address OAGB technical aspects using a national survey completed by members of the Israeli Society of Metabolic and Bariatric Surgery (ISMBS).

**Materials and Methods:**

An online-survey composed of a 17-item–based questionnaire was sent to ISMBS members. All responses were collected and analyzed.

**Results:**

A total of 47/64 (73.4%) ISMBS members participated in the survey. Most surgeons (74.5%) had > 10 years of MBS experience, and most (61.7%) performed > 100 MBS/year. The majority (78.7%) perform OAGB as their most common procedure. Most surgeons fashion a 10–15-cm pouch and use a 36-Fr bougie, (57.4% and 38.3%). Regarding bowel length measurement, 70% use visual estimation and 10.6% routinely measure total small bowel length (TSBL). The most common reasons for creating a longer biliopancreatic limb (BPL) were high body mass index (BMI) and revisional surgery (83.3% and 66%, respectively). In a primary procedure of a patient with a BMI = 40, most (76.6%) would create a 150–200-cm BPL. In patients with a BMI > 50 or revisional cases, most (70.2% and 68.0%) would create a 175–225-cm BPL.

**Conclusion:**

OAGB is the most prevalent MBS performed in Israel. This survey showed common preferences and variations among ISMBS members. Further studies are needed in order to standardize and build a consensus on OAGB technique.

**Supplementary Information:**

The online version contains supplementary material available at 10.1007/s11695-024-07223-3.

## Introduction

In the ongoing search after the ideal metabolic and bariatric surgery (MBS), one anastomosis gastric bypass (OAGB) is gaining popularity worldwide [1]. The combination of technical simplicity, a high safety index and excellent outcomes make it an appealing choice for both patients and surgeons [2].

While cautiously adopted in other regions of the world, OAGB has become the procedure of choice in Israel, comprising nearly 70% of the total number of MBS [3]. Disappointing long-term outcomes of the sleeve gastrectomy (SG)—the previous procedure of choice, and the more technically challenging Roux-en-Y gastric bypass (RYGB) both contributed to this shift towards OAGB [4].

Although usually relatively simple to perform, some are still hesitant to adopt it—mainly criticizing side effects such as bile reflux and significant malabsorption sometimes leading to severe diarrhea, malnutrition, and hypoalbuminemia [5]. Long-term studies evaluating OAGB have shown a reasonable balance between its efficacy and undesirable sequelae [6–9].

It seems there is a huge variability in surgical technique regarding OAGB [10]. The IFSO update position statement on OAGB summarizes the different operative techniques but does not conclude the “correct” way to perform this procedure [11]. Many questions arise when performing OAGB: pouch size—should it be loose and offer less restriction, or should it be narrow to reduce acid formation? Anastomotic size—do we aim for a narrow anastomosis to reduce dumping and improve restriction, or is it better to perform a wide anastomosis for easy emptying of the pouch and less stenosis? What is the ideal length of the biliopancreatic limb (BPL)—150 cm? 200 cm? Or maybe the ideal length depends on total bowel length? Or on patient body mass index (BMI)? And should the bowel length be measured? Is a rough visual estimation sufficient or should a more precise method be used?

With nearly 8200 bariatric surgeries performed in Israel in 2021, 5635 were OAGB (68.2%). Nearly 18,000 OAGB were performed nationally in the last 4 years. Building on this vast experience, the aim of this study was to describe the common practices and variations of surgeons performing OAGB in Israel. Analyzing this information may be helpful in the attempt to standardize OAGB for favorable outcomes with minimal complications and side effects.

## Methods

An online questionnaire-based survey was conceived and prepared by the Bariatric Unit of the Division of General Surgery in the Tel-Aviv Sourasky Medical Center. The survey was sent through a mailing list and social media to the members of Israeli Society of Metabolic and Bariatric Surgery (ISMBS) on January 10, 2024, and was closed for analysis after 30 days. The survey was voluntary and confidential. It was composed of 17 questions that address technical aspects of OAGB and was divided into three parts: (1) gastric pouch characteristics, (2) small bowel measurement technique, and (3) factors that impact decision of bypass length. The questionnaire was written in Hebrew, but a translated English version can be seen in the electronic supplementary material.

All questions were multiple-choice based, some of which were multi-select. All responses were recorded. Data are reported using numbers (percentages) and analyses are presented in a descriptive and/or graphical manner.

## Results

### Respondents

The questionnaire was sent to the ISMBS members on January 17, 2024, and was open for response for 30 days. The association has 64 active members, and 47 members (73.4%) responded. The characteristics of respondents can be seen in Table 1. Nearly three quarters of respondents (*n* = 35, 74.5%) had more than 10 years of MBS experience and nearly two-thirds of respondents (*n* = 29, 61.7%) perform more than 100 MBS procedures per year. For the vast majority of surgeons, the most commonly performed MBS procedure was OAGB (*N* = 37, 78.7%), with SG and RYGB being the most commonly performed MBS procedure for a minority of surgeons (*n* = 6, 12.8% and *n* = 4, 8.5%, respectfully).

**Table 1:** Characteristics of respondents

Characteristic:	Number (percent):
Years of MBS experience	
< 3	3 (6.4%)
3–10	9 (19.1%)
> 10	35 (74.5%)
Volume of MBS procedures per year	
≤ 50	6 (12.8%)
50–100	11 (23.4%)
100–200	15 (32.0%)
> 200	14 (29.8%)
NA	1 (2.1%)
Most commonly performed MBS	
OAGB	37 (78.7%)
SG	6 (12.8%)
RYGB	4 (8.5%)

*MBS* Metabolic and bariatric surgery, *NA* not available, *OAGB* one anastomosis gastric bypass, *SG* sleeve gastrectomy, *RYGB* Roux-en-Y gastric bypass

### Procedural Technique

**Fig. 1:**
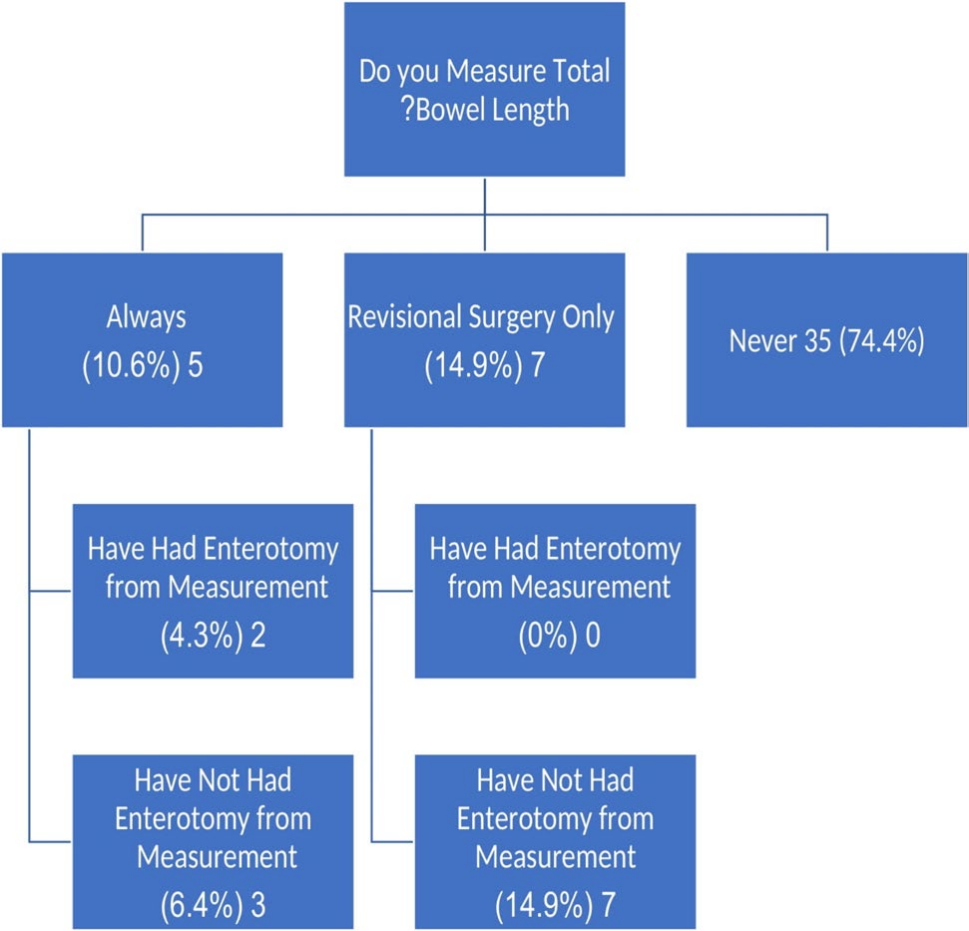
Practice and experience with total small bowel length measurements

The procedural technique of the responding surgeons can be seen in Table 2. When performing OAGB, the vast majority of surgeons create a gastric pouch of 10–20 cm (*N* = 42, 89.3%), most opting for a pouch of 10–15 cm (*n* = 27, 57.4%). Among respondents, 38 (80.8%) use a bougie of 32–36 Fr, with a 36-Fr bougie being the most commonly chosen size (*n* = 18, 38.3%). Only two respondents use a 40-Fr bougie (4.3%), and only one respondent (2.1%) does not use a bougie at all. For a standard procedure (defined in this questionnaire as a primary OAGB in a 40-year-old patient with BMI of 40 kg/m^2^), more than three quarters of surgeons (*n* = 36, 76.6%) fashion a BPL measuring 150–200 cm. Half of this sub-group create a 150–175-cm BPL (*n* = 18, 38.3%) and the other half create a 176–200-cm BPL (*n* = 18, 38.3%). Three respondents (6.4%) tailor the length of the BPL to a proportion of the total bowel length of the specific patient undergoing OAGB. Most surgeons (*n* = 33, 70.2%) use visual estimation as the method to measure bowel length, with a minority (*n* = 14, 29.8%) using the aperture of the grasper for measurement, and none utilizing an intra-abdominal ruler pr nylon-tape. Only five (10.6%) surgeons measure the total bowel length in every OAGB procedure, and additional seven surgeons (14.9%) measure the total small bowel length only during revisional procedures. Most surgeons (*n* = 35, 74.4%) never measure the total small bowel length during OAGB (Fig. 1). Only two of 12 respondents that measure the total bowel length (16.6% of this sub-group and 4.3% of all respondents), either as standard practice or during revision, have experienced an enterotomy during measurement (Fig. 1).

**Table 2:** Procedural technique as reported by respondents

Characteristic:	Number (percent):
Length of pouch (cm)	
6–9	4 (8.5%)
10–15	27 (57.4%)
16–20	15 (31.9%)
> 20	1 (2.1%)
Diameter of bougie (FR)	
32	8 (17.0%)
34	12 (25.5%)
36	18 (38.3%)
38	6 (12.8%)
40	2 (4.3%)
Does not use bougie	1 (2.1%)
Biliopancreatic limb length in primary surgery* (cm)	
< 150	6 (12.8%)
150–175	18 (38.3%)
176–200	18 (38.3%)
201–225	2 (4.3%)
< 40% total bowel length	2 (4.3%)
40–50% total bowel length	1 (2.1%)
Technique for bowel length measurement	
Visual estimation	33 (70.2%)
Comparison to grasper aperture	14 (29.8%)
Intra-abdominal ruler	0 (0%)

A 40 year old patient with a body mass index of 40 kg/m^2^

### Tailoring Procedural Technique to the Clinical Scenario

**Fig. 2:**
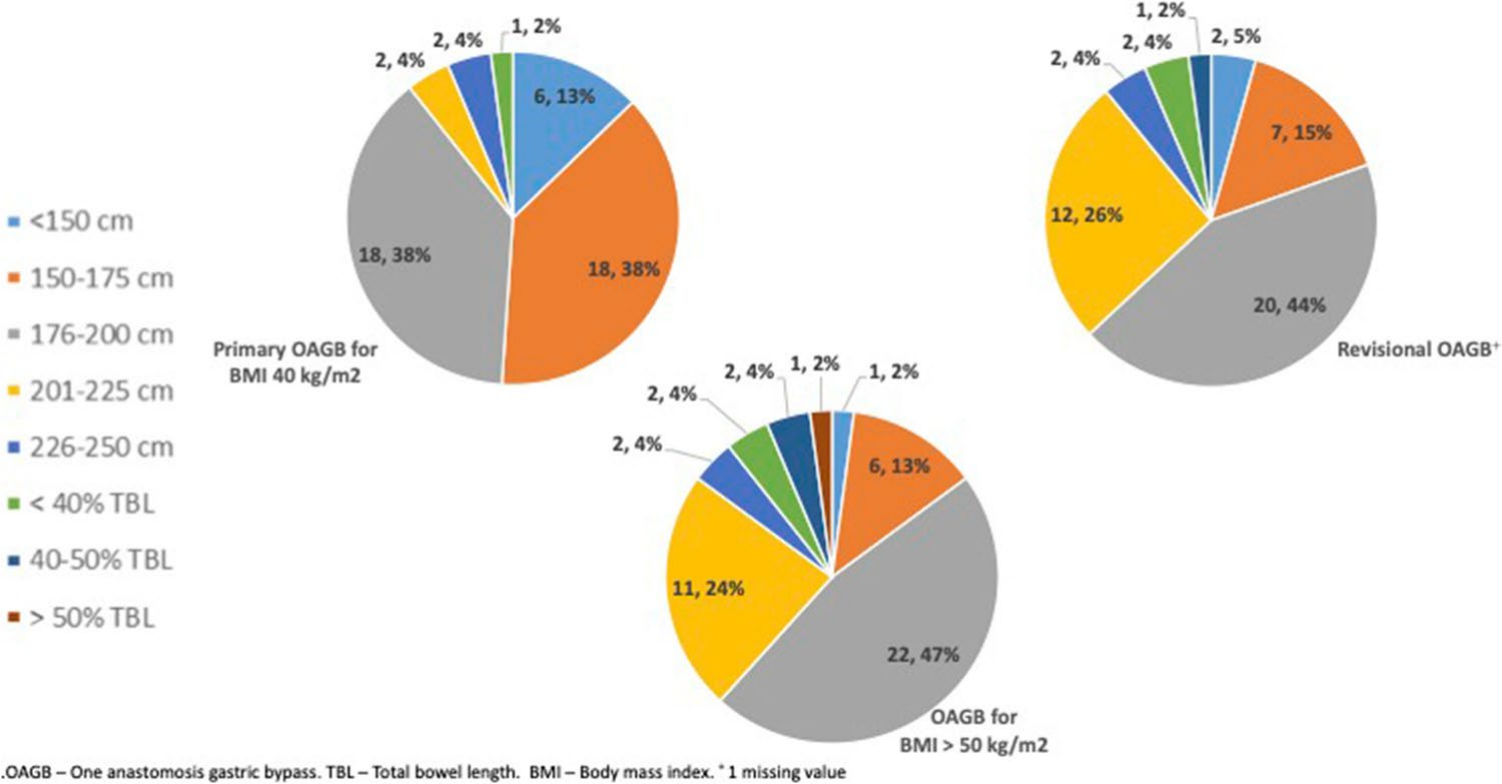
Choice of biliopancreatic limb length based on clinical scenario

Respondents were asked which clinical presentations would make them change the length of the BPL (Table 3). The surgeons could choose more than one response. The most common reasons for changing the length of the BPL length included, in decreasing order—BMI (*n* = 39, 83.0%), revisional surgery (*n* = 31, 66.0%), obesity associated medical problems (*n* = 15, 31.9%), age (*n* = 13, 27.7%), height (*N* = 4, 8.5%), and gender (*n* = 3, 6.4%). Five respondents (10.6%) did not choose any of the above, and it is our understanding that they perform a BPL of constant length regardless of the clinical scenario. Figure 2 shows the proportion of surgeons choosing BPL length for three different clinical scenarios: 40-year-old patient with BMI of 40 kg/m^2^, a revisional procedure, and a patient with a BMI > 50 kg/m^2^. In the last two scenarios, many surgeons chose to make a longer BPL than in the first scenario. For example, in the first scenario, 6 surgeons (12.8%) would create a short BPL of up to 150 cm, whereas only two surgeons (4.3%) would create a BPL of this length in a revisional procedure and only one surgeon (2.1%) would create a BPL of this length in a patient with BMI of 50 kg/m^2^. In the first (standard) scenario, only two surgeons (4.3%) would create a long BPL of 201–225 cm, but 12 (25.5%) and 11 surgeons (23.4%) would create a BPL of this length in a revisional procedure and a high BMI patient, respectfully (Fig. 2).

**Table 3:** Patient factors that would cause respondents to tailor/change the biliopancreatic limb length

What would make you change your standard biliopancreatic limb length? *	*N* (percent)
BMI	39 (83.0%)
Revisional surgery	31 (66.0%)
Obesity-associated medical problems	15 (31.9%)
Age	13 (27.7%)
Height	4 (8.5%)
Gender	3 (6.4%)
The limb should be of constant length	5 (10.6%)

^*^Respondents could choose more than one response. *BMI* Body mass index

## Discussion

The results of this study report the technical considerations and choices of surgeons from the ISMBS when performing OAGB. The survey was completed by a large percentage of the ISMBS members, and a majority of respondents were high volume surgeons with at least a decade of experience performing MBS. Most ISMBS surgeons who completed the questionnaire create a pouch of 10–20 cm, and utilize a bougie of 32–36 Fr. In a standard procedure, most respondents create a BPL limb of 150–200 cm, although some surgeons tailor the length of the limb to a proportion of the total small bowel length. One of the advantages of OAGB is the ability to tailor the operation to the clinical scenario at hand. Many surgeons among the ISMBS respondents change the length of BPL, creating a longer limb during revisional surgery and when treating patients with higher BMI.

OAGB, first described by Robert Rutledge in the beginning of the twenty-first century [12], has been reported in many studies throughout the years, and in the last position statement by the International Federation for the Surgery of Obesity and Metabolic disorders (IFSO), OAGB has been reported to have satisfactory outcomes as a primary procedure and was recognized as a bariatric and metabolic procedure [13]. In addition, the American Society of Metabolic and Bariatric Surgeons (ASMBS) posted a statement endorsing OAGB as a metabolic and bariatric procedure in light of many studies showing favorable short- and long-term outcomes in studies conducted outside the United States [14]. In Israel, OAGB has become more and more common in the last decade, and according to our survey, it is the most commonly performed procedure for 78.7% of ISMBS surgeons. To our knowledge and according to the national bariatric registry, Israel is the only country reporting OAGB to be the most common MBS performed [3]. We believe that this trend will expand worldwide and OAGB will become far more common among other countries in the next decade.

OAGB technique varies widely, and the aim of this study was to report the ISMBS technical considerations on OAGB which may aid in further standardization of this procedure. The length of the gastric pouch in OAGB varies among many studies as reported in the IFSO statement in 2018 [15]. Despite these variations, nearly all studies report a relatively long pouch with dissection beginning at the level or just below the Crow’s foot on the lesser curvature and the use of a 36-Fr bougie for pouch calibration. Similarly, in the current study, nearly 90% of respondents aim to construct a pouch of 10–20 cm (57.4% 10–15 cm, 31.9% 16–20 cm), and most use a 36-Fr bougie. The literature regarding the actual pouch size performed is limited, and most studies recommend a long pouch and relate to anatomical landmarks such as beginning the dissection at/or below the crow’s foot and do not routinely measure the actual pouch size as written in the last IFSO consensus statement on OAGB [11]. The recommended pouch size in other statement studies and reviews is at least 15 cm [14, 16] which is in the range of most participant’s responses. The gastric pouch should generally be as long as possible, and dissection should begin below the level of the crow’s foot. There are many advantages to constructing a long and narrow pouch—a long gastric pouch provides a rich vascularity for the gastric tissue and anastomotic site which is essential for tissue healing and rich oxygenation [17]. A narrow pouch may provide an additional restrictive effect for the patient and is probably associated with less acid secretion which may reduce the risk of marginal ulcers [18]. Ayuso et al. [19] retrospectively analyzed patients undergoing RYGB and showed that on computed tomography (CT) volumetric analysis, a larger gastric pouch was associated with marginal ulcers following RYGB. There are no similar volumetric trials in OAGB defining the ideal pouch length of OAGB with regards to all the aforementioned issues. In addition, OAGB can be associated with bile reflux and pouch size can play a role in its development; Saarinen et al. [5] evaluated bile reflux in patients after OAGB with a 15-cm pouch and found bile reflux in 31.6% at 6 months postoperatively in bile reflux scintigraphy. However, the cohort was relatively small (*n* = 49) with a short follow-up (6 months). There are no studies assessing pouch size and its impact on bile reflux.

Measurement of small bowel length has always been an area of controversy as reported studies show significant inaccuracy of measurements which can reach > 15% deviation [20]. Musie et al. [21] showed that the method of measurement plays an important role and that utilizing rigid tools as a ruler can underestimate the TSBL and Tacchino et al. [22] reported that a fully stretched bowel could increase the measurements to by more than 100 cm. Most respondents in our study (70%) use a rough visual estimation only, and we believe this is the main practice among many surgeons worldwide despite available data on its inaccuracy. This is mainly in attempt to shorten the length of the procedure, to avoid small bowel injury, and a possible low yield when using more sophisticated methods for measurements.

The length of the BPL impacts weight loss and resolution of obesity associated medical problems and the recommended BPL length for OAGB should be at least 150 cm with a desirable length of up to 200 cm length [23] which is similar to results of our survey showing nearly 80% perform a BPL length of 150–200 cm. The metabolic effect of a longer BPL is associated with increased incretin secretions, increased systemic bile acids that can result in greater weight loss, and there may be a favorable effect on the microbiome [14]. Despite the positive effects of a longer BPL length, it has its downsides including increased risk of nutritional deficiencies. In a systematic review by Felsenreich et al. [24], it was reported that the differences in BPL length tend to impact nutritional deficiencies more than weight loss and resolution of medical problems, and it is also recommended to measure the entire bowel length in cases when performing more than 200-cm BPL to reduce the risk of malnutrition. In our study, only 10% of surgeons measure the total small bowel length routinely, and only 15% measure the total small bowel length in revisional cases, of which 4% experience at least one complication when routinely measuring the total small bowel length. Interestingly, Soong et al. [25] reported that in comparison to a tailored bypass only, a technique of tailoring to patient characteristics and according to the total small bowel length measurement while keeping a 400-cm common channel is associated with a significantly lower rate of nutritional complications. Routine measurement of the total small bowel length may produce a protective effect in 1–2% of the population with an extremely short small bowel, preventing devastating malnutrition. There may be a benefit for total small bowel length measurement in OAGB; however, robust data assessing this topic are lacking.

Most respondents tailor the BPL length according to the patients’ characteristics—most commonly to the patients’ BMI and to revisional indications (80% and 66%, respectively). When compared to a 150 cm, a 200-cm BPL length was shown to be associated with increased total weight loss in patients undergoing OAGB in a systematic review and meta-analysis published by Salman et al. [26]. In an additional review by Parmar et al. [27] evaluating patients with a BMI > 50 kg/m^2^ undergoing OAGB, the median BPL length was 280 cm, the mean percentage of excess weight loss and BMI at a follow-up of 5 years was 90.75% and 27.7 kg/m^2^, respectively, and there were no reports of malnutrition. Moreover, in a narrative review by Kermansaravi et al. [16], it was suggested to perform a BPL length of at least 200 cm for patients with a BMI > 50 kg/m^2^. In our study, BMI was the most significant factor influencing the BPL length, but other factors should also be taken into consideration when tailoring the length (such as revisional surgery, height, gender, age, and associated medical problems).

Routine measurement of total small bowel length and tailoring the limb accordingly may be of benefit, and as reported by Soong et al. [25], it was associated with reduced nutritional complications. Komaei et al. [28] reported similarly that tailoring the limb according to the total small bowel length when compared to a fixed 200 cm is associated with similar weight loss results and significantly lower nutritional deficiencies at 1 year follow-up. Interestingly, in this study, the mean BPL length in the tailored group was 250 cm, and only in one patient (1/32), the BPL limb was < 200 cm (160 cm). This study is underpowered due to its small cohort (*n* = 64), and selection bias could occur due to the retrospective nature and the results. According to the latest IFSO and ASMBS position statement on OAGB [11, 14], tailoring the biliopancreatic limb according to the total small bowel length is not routinely performed. To our knowledge, there are no published randomized clinical trials, and there is an ongoing randomized trial addressing this topic [29]. Adjusting the BPL length according to total small bowel length may be of benefit due to the great variability of the total small bowel length in different patients.

To summarize, OAGB is the most commonly performed MBS in Israel, and there is a high volume of OAGB performed nationally by MBS surgeons. There are several technical considerations and preferences which were shown to be similar among ISMBS surgeons; however, there are still some discrepancies and variations among this group. We call for more surgeons worldwide to report their experience in OAGB to further help standardize the OAGB technique.

This study has several limitations; the number of respondents is relatively low (n = 47). Data is based on surgeons’ experience and technical aspects with no reported data on short term complications, weight loss outcomes, or resolution of obesity-related disease. In addition, there may not always be a correlation between high volume and quality of surgery. Despite this, the number of respondents consists of a major percentage of ISMBS members, and it reports the technical experience of veteran and high-volume MBS surgeons in a country where OAGB is the most commonly performed MBS.

## Conclusion

In conclusion, OAGB is the most commonly performed MBS in Israel. Despite many similarities in this ISMBS member survey, there were still several variations in OAGB technical considerations. BMI and revisional surgery were considered the most important factors when tailoring BPL length. Additional studies addressing these aspects could help in standardizing OAGB procedure.

## Supplementary Information

Below is the link to the electronic supplementary material.

Supplementary file1 (DOCX 17 KB)
