# Correction: Technical Considerations in One Anastomosis Gastric Bypass—the Israeli Society of Metabolic and Bariatric Surgery Experience

**DOI:** 10.1007/s11695-024-07541-6

**Published:** 2024-10-12

**Authors:** Adam Abu-Abeid, Jonathan Benjamin Yuval, Andrei Keidar, Eran Nizri, Guy Lahat, Shai Meron Eldar, the ISMBS collaborative group

**Affiliations:** Division of General Surgery, Tel Aviv Sourasky Medical Center, Sackler Faculty of Medicine, Tel Aviv University, 6 Weizman Street, 64230906 Tel Aviv, Israel

**Correction: Obesity Surgery (2024) 34:2356–2362**

10.1007/s11695-024-07223-3

The original article has been updated to add author tagging for the members of the collaborative group listed in the Acknowledgements section.

