# Supplementary material for: Technical Considerations in One Anastomosis Gastric Bypass—the Israeli Society of Metabolic and Bariatric Surgery Experience
Source: Obes Surg. 2024 Apr 23;34(7):2356–62. doi: 10.1007/s11695-024-07223-3 (PMC11217076; doi:10.1007/s11695-024-07223-3)
Supplement: Supplementary file 1 — Supplementary file1 (DOCX 17 KB) [file 11695_2024_7223_MOESM1_ESM.docx]

Supplementary table 1 – Questionnaire on OAGB technical considerations for ISMBS members

| Question | Answer |
| --- | --- |
| 1.How many years do you practice bariatric surgery? | 1. <3 years 2. 3-10 years 3. >10 years |
| 2. How many bariatric procedures do you perform annually? | a. up to 50  b. 50-100  c. 100-200  d. >200 |
| 3. Which bariatric procedure do you perform mostly? | a. Sleeve Gastrectomy  b. One Anastomosis Gastric Bypass (OAGB)  c. Roux-en-Y Gastric Bypass  d. Single anastomosis duodeno-ileal bypass  e. Other |
| 4. What is the pouch length you perform in OAGB? | a. <5 cm  b. 6-9 cm  c. 10-15 cm  d. 16-20 cm  e. >20 cm |
| 5. What bougie size do you use in OAGB? | a. 32 Fr  b. 34 Fr  c. 36 Fr  d. 38 Fr  e. 40 Fr  f. other |
| 6. What is the anastomotic length you perform in OAGB? | a. 30 mm  b. 30-45 mm  c. 45-60 mm  d. other |
| 7. How do you measure the bypassed bowel length? | a. Visual estimation  b. Comparison to grasper aperture  c. Intra-abdominal ruler/nylon tape  d. other |
| 8. Do you measure total small bowel length during OAGB? | a. No  b. Always  c. Only in revisional cases |
| 9. If you measure the total small bowel length, did you ever experience a complication from this (such as enterotomy)? | a. Yes  b.No |
| 10. If you do not measure routinely total small bowel length, what is the reason for that? | a. I do not think it is of significance  b. The disadvantages outweigh the advantages such as increased operative time and possible bowel injury.  c. I have tried this method before but I stopped (please provide a reason in comment)  d. other |
| 11. If you **do not measure** the total small bowel length, which characteristics will influence the length of the biliopancreatic limb you will perform? (More than one answer can be provided) | a. Revisional surgery  b. Height  c. BMI  d. Age  e. Associated medical problem  f. Gender  g. The limb should be of constant size |
| 12. If you **measure** the total small bowel length, which characteristics will influence the length of the biliopancreatic limb you will perform? (More than one answer can be provided) | a. Revisional surgery  b. Height  c. BMI  d. Age  e. Associated medical problem  f. Gender  g. The limb should be of constant size |
| 13. In a primary standard OAGB, 40-year-old patient with BMI of 40. What is the length of the biliopancreatic limb you perform? | a. < 150 cm  b. 150-175 cm  c. 175-200 cm  d. 200-225 cm  e. 225-250 cm  f. > 250 cm  g. < 40% Total Bowel Length  h. 40-50% Total Bowel Length |
| 14. In revisional OAGB due to failure of previous bariatric procedure. What is the length of the biliopancreatic limb you perform? | a. < 150 cm  b. 150-175 cm  c. 175-200 cm  d. 200-225 cm  e. 225-250 cm  f. > 250 cm  g. < 40% Total Bowel Length  h. 40-50% Total Bowel Length |
| 15. In a patient with BMI > 50, What is the length of the biliopancreatic limb you perform? | a. < 150 cm  b. 150-175 cm  c. 175-200 cm  d. 200-225 cm  e. 225-250 cm  f. > 250 cm  g. < 40% Total Bowel Length  h. 40-50% Total Bowel Length |
| 16. If you would like to be a collaborative author please provide your full name |  |
| 17. If you would like to be a collaborative author please provide your email address |  |
